# Review of Performance Improvement of a Noninvasive Brain-computer Interface in Communication and Motor Control for Clinical Applications

**DOI:** 10.14789/jmj.JMJ23-0011-R

**Published:** 2023-08-25

**Authors:** YUYA SAITO, KOJI KAMAGATA, TOSHIAKI AKASHI, AKIHIKO WADA, KEIGO SHIMOJI, MASAAKI HORI, MASARU KUWABARA, RYOTA KANAI, SHIGEKI AOKI

**Affiliations:** 1 Department of Radiology, Juntendo University Graduate School of Medicine, Tokyo, Japan; 1 Department of Radiology, Juntendo University Graduate School of Medicine, Tokyo, Japan; 2 Department of Radiology, Toho University Omori Medical Center, Tokyo, Japan; 2 Department of Radiology, Toho University Omori Medical Center, Tokyo, Japan; 3 Araya Inc., Tokyo, Japan; 3 Araya Inc., Tokyo, Japan

**Keywords:** brain-computer interface, medicine, deep learning, machine learning, data augmentation

## Abstract

Brain-computer interfaces (BCI) enable direct communication between the brain and a computer or other external devices. They can extend a person's degree of freedom by either strengthening or substituting the human peripheral working capacity. Moreover, their potential clinical applications in medical fields include rehabilitation, affective computing, communication, and control. Over the last decade, noninvasive BCI systems such as electroencephalogram (EEG) have progressed from simple statistical models to deep learning models, with performance improvement over time and enhanced computational power. However, numerous challenges pertaining to the clinical use of BCI systems remain, e.g., the lack of sufficient data to learn more possible features for robust and reliable classification. However, compared with fields such as computer vision and speech recognition, the training samples in the medical BCI field are limited as they target patients who face difficulty generating EEG data compared with healthy control. Because deep learning models incorporate several parameters, they require considerably more data than other conventional methods. Thus, deep learning models have not been thoroughly leveraged in medical BCI. This study summarizes the state-of-the-art progress of the BCI system over the last decade, highlighting critical challenges and solutions.

## Introduction

Brain-computer interface (BCI) can connect the brain and the external world by identifying brain activity and translating it into messages or commands, without depending on normal peripheral nerves and muscles^1)^. In particular, electroencephalogram (EEG) as noninvasive BCI has been developed for clinical purposes. For instance, EEG- based BCI has potential applications in the rehabilitation of patients suffering from stroke^2)^, tactile system of communication and control options for patients with impairments of eye movements or vision^3)^, and prognosis for patients with cognitive motor dissociation^4)^. However, challenges and limitations of BCI systems in clinical use still prevail.

Herein, we focus on BCI studies published between 2011 and 2021 to investigate the most recent trends in BCI research in the area of communication and control for clinical applications, such as the extraction of action intentions and translation into electrical commands and the monitoring of human physiology in patients with motor disabilities. Further, this study discusses the challenges and limitations of BCI systems along with solutions to these issues. This study also suggests future research for introducing BCI systems to medical fields.

## Methods

In this mini-review, we investigate papers published in the past 10 years (January 2011-January 2021) that have been cited more than 100 times to refine search articles with high impact according to the Web of Science database. Papers with headings, abstracts, or keywords including the phrase “brain-computer interface” have been selected, and it has been ensured that the type of document is “article” rather than proceedings paper or review.

## Performance Evaluation

Although many evaluation metrics have been used to quantify performance in the BCI field, the most common metric is classification accuracy^5, 6)^, which allows the measurement of the number of trials classified correctly as a percentage of all trials. Therefore, the accuracy obtained for each subject is used to evaluate existing BCI systems in this study. In a previous report, a BCI system with an accuracy of <70% was deemed unacceptable, whereas a BCI system with an accuracy of >75% was deemed successful^7)^. Based on this report, the accuracy criteria were defined to compare existing BCIs (Table 1) in the present study.

**Table 1:** BCI performance summary

Reference	Year	Algorithms	Signal type	Accuracy (%)	Performance	Online/Offline
8)	2016	Hamming window, STFT, PCA, Linear regression	Translate thinking actions into electrical commands	74.6	Fair	Online
9)	2014	FFT, SLIC	Translate thinking actions into electrical commands	70.0	Fair	Offline
10)	2015	Theta spectra, threshold	Translate the physiological state into music selection	71.4	Fair	Online
11)	2017	Band-pass filter, average power, temporal correlation	Translate thinking actions into hand movements	70.0	Fair	Offline
12)	2016	DWT, SVM	Translate thinking actions into hand movements	82.1	Good	Offline
13)	2019	SCSSP, MI, LDA, SVM	Translate thinking actions into hand movements	81.9	Good	Offline
14)	2018	CNN	Translate thinking actions into hand movements	70.0	Fair	Online
15)	2019	CNN	Translate thinking actions into hand movements	80.5	Good	Offline
16)	2020	LSTM	Translate thinking actions into hand movements	97.6	Good	Offline

Abbreviation: STFT, short-time Fourier transform; PCA, primary component analysis; FFT, fast Fourier transform; SLIC, stimulus-locked inter-trace correlation; DWT, discrete wavelet transform; SVM, support vector machine; SCSSP, Separable Common Spatiospectral Pattern; MI, mutual information; LDA, linear discriminant analysis; CNN, convolutional neural network; LSTM, long short-term memory.

BCI systems can be validated both offline and online. An offline approach validates a BCI system by using a dataset that has already been collected and shared by a few research groups. In most cases, the validation of a BCI system begins with identifying appropriate signal processing techniques offline. A subsequent online analysis is used to validate the performance of the BCI system for extracting and classifying trials from real data. Real-time embedded EEG-based brain-computer interface can be used for controlling electrical devices using EEG signals, which is an online approach.

## Review of BCI Applications

This section introduces the selected papers, enabling a comprehensive understanding of the trend in the history and evolution of BCI systems to date in terms of the preprocessing and classifier models based on EEG signals.

EEG signals were developed^8)^ to extract thinking actions and translate them into electrical commands to develop an embedded BCI system that can be used to control electrical devices. The input EEG signals were filtered using an EEG filter block that extracts essential features and were converted into electric commands to activate the corresponding actions intended by the patient with severe motor disorders. The classification accuracy of translation into electrical commands was 74.6% based on linear regression via the online approach.

For improved biopotential acquisition and processing, an autonomous embedded BCI system was developed^9)^ based on an ARM9 processor that can port a real-time operating system for visual- evoked potentials. The results show that this application recovered visual evoked potentials using fast Fourier transform (FFT) by extracting frequency-domain features from BCI signals and stimulus-locked interlace correlation (SLIC); thus, a classification method based on EEG signals in the time domain was proposed. Additionally, the classification accuracy was found to be 70% upon examination of the steady-state visual-evoked potential phase-locking and time-locking in terms of the stimulus properties via the offline approach.

Further, a BCI-based smart multimedia controller was introduced^10)^, which can select music in different situations according to the user's physiological state. The multimedia platform in that study comprised an easily available commercial mobile tablet and a wireless multichannel EEG acquisition module designed for real-time EEG monitoring. A smart multimedia control program built into the multimedia platform was successfully developed to analyze the user's EEG and select music based on the user's physiological state. The experimental results show a classification accuracy of 71.4% via the online approach. The wireless multichannel EEG acquisition module can easily communicate with any type of commercial tablet via Bluetooth, thereby increasing acceptability among a large user demographic. Therefore, this BCI system is versatile and can be used on different evoked potential scenarios, such as medical brain-computer interfaces, while satisfying the strict real-time constraints that they impose.

However, BCI systems developed in previous studies were expensive and had limited portability; thus, the applications of BCI systems were limited. To address the lack of portability and high cost issues of BCI systems, a portable, low-cost BCI was developed and compared with a conventional BCI^11)^. Specifically, five subjects were tested who were cued to alternate between hand opening/closing or were motionless while the BCI decoded their state of movement in real-time. The performance in each trial was defined as the temporal correlation between the cues and the decoded states. The results show that the EEG data acquired using the proposed and conventional BCIs were highly correlated (*ρ* = 0.79). The decoding performances, obtained using linear discriminant analysis, of the proposed and conventional BCIs were 70% and 68% in the offline approach, respectively, when averaged across trials and subjects; thus, the performances were not significantly different from each other.

Although previous studies used conventional and statistical classification models^12)^, introduced machine learning models such as support vector machines (SVM) for BCI systems. There is a background that the computer system had enough computational resources for fully embedded BCI systems. The classification result of a 2-class motor imagery paradigm was 82.1% using the SVM classifier and minimal processing time (0.11 s) in the offline approach on the embedded device in the experimental result, allowing the development of a portable, low-cost, and trustworthy system. Similarly^13)^, proposed a BCI system based on an SVM classifier. The proposed method includes statistical learning methods such as mutual information (MI), LDA, and SVM and applies the separable common spatiospectral pattern (SCSSP) method to extract features to design an accurate algorithm. The classification accuracy of a two-class motor imagery paradigm was 81.9% in the offline approach. The proposed BCI system achieves not only excellent recognition accuracy but also remarkable implementation efficiency in terms of portability, power, time, and cost.

Recently, deep learning techniques have been employed to improve the performance of BCI systems. Deep learning models can express more subtle and complex features in EEG signals than traditional machine learning techniques. Therefore, deep learning models are expected to provide more accurate predictions. A BCI system was^14)^ developed using the convolutional neural network (CNN) EEGNet, a compact version of the existing CNN, for feature extraction and classification of motor imagery. As EEGNet is based on depthwise convolutional and separable convolution, the number of parameters in EEGNet is reduced. The EEG signals were processed as a series of multichannel images in a continuous-time domain showing the energy changes in the cerebral cortex during motor imagery of the subjects. The classification accuracy reached approximately 70.0%. To improve this system, a field-programmable gate array (FPGA) accelerator system^15)^ was proposed, which combines both flexibility and reconfigurability of different CNN structures. Applying the synchronous dataflow model to an embedded system and configuring the intellectual property cores of each layer separately, a 16-bit fixed-point CNN was finally used for EEG classification. The classification accuracy reached 80.5%, and the proposed design was approximately eight times faster and more efficient than the conventional BCI system in terms of execution time and power consumption. In another study, a BCI system with deep learning specialized in time series data called long short-term memory (LSTM) was proposed^16)^ to improve the quality of life for patients with motor disabilities. The proposed BCI system used multiple convolutional LSTM and fully-connected layers to decode EEG signals to maximize human intention recognition accuracy. The classification accuracy for a two-class motor imagery paradigm was 97.6% in the offline evaluation. Moreover, the proposed model reduces power consumption by 62.7% and improves the throughout power (W) by 168% compared with the previous models using central processing units, graphics processing units, field programmable gate arrays, application-specific integrated circuits, resistive random access memories, and photonic neural network accelerators.

Therefore, the BCI system has evolved from a simple statistical model to a deep learning model, and its performance has improved with time and the enhancement of computational power in computers.

## Challenges and Limitations

Many BCI achievements in the SLIC application field have been reported. Furthermore, benchmark datasets can achieve state-of-the-art performance with high classification accuracy. However, most achievements have been validated using only the offline rather than the online approach. The performance in EEG trial classification obtained via the offline approach significantly decreases compared with that of the online approach^17)^. For example, BCI was developed^18)^, enabling a controlled functional electrical stimulation (FES) based on EEG owing to stroke for re-establishing foot dorsiflexion. The study generated a prediction model based on approximate information discriminant analysis to classify EEG data into either “idling” or “dorsiflexion” stages; this information was subsequently used to control an FES device to elicit effective foot dorsiflexion. Although the average offline classification was 98.8%, the average online classification was 50%^19)^. using two types of oddball paradigms, including the silk-stim paradigm (SSP) and linen-stim paradigm (LSP). The offline classification accuracies based on Bayesian linear discriminant analysis of the two paradigms for SSP and LSP were 64.5% and 75.5%, respectively, whereas the online classification accuracies were 50.0% and 53.0%, respectively. The steady-state visual-evoked potential-based BCI performance investigated under different perturbations^20)^. The subjects focused on one of the four circles and provided feedback on the correctness of the classification under four conditions that were randomized across the subjects: *Control* (no perturbation), *Speaking* (counting loudly and repeatedly from 1 to 10), *Thinking* (mentally counting repeatedly from 1 to 10), and *Listening* (listening to verbal counting from 1 to 10). Although the offline mean classification accuracy using decision tree was 97.0%, the online mean classification accuracy was 83.0%.

Therefore, the accuracy of BCI systems decreases by approximately 20.0%-50.0% during the validation of the EEG signal processing chain based on the online approach. This could be attributed to the lack of sufficient data for rendering the classifier more robust and reliable. Compared with that in computer vision and speech recognition fields, the training samples in the medical BCI field are limited, as patients whose EEG data collections are limited compared with healthy controls are targeted. Additionally, the deep learning models require much more data than other conventional methods^21, 22)^ and cannot fully utilize the potential of the deep learning model in the medical BCI field.

## Solution for the Problem

To overcome the prevalent data deficiency problems, novel approaches have been proposed to generate artificial brain signals and improve the performance of BCI systems. The first method was proposed by Fabien, which was implemented using mixing signal segmentation in the time domain^23)^. This approach can significantly increase classification accuracy even for small training datasets. However, this approach poses the limitation of causing inadequate high-frequency noise at the boundary between two different segmentations. To overcome this problem, artificial EEG signal generation methods based on time-frequency representation (TFR) and analogy methods were proposed^24)^. The aforementioned approach only considered the temporal features of EEG signals, not the frequency features. Therefore, an empirical mode decomposition method was proposed^25)^ to consider the features in the temporal and frequency domains. To further improve the classification accuracy, the differential entropy feature was used to generate more EEG signals, and this method could significantly improve the performance of deep learning models (LeNet and ResNet)^26)^.

Although all previous methods for generating artificial EEG signals were based on a combination of the features of raw EEG signals in different trials, various novel deep learning methods have recently been proposed to generate artificial EEG signals from the probability distribution and deep learning perspective rather than physically combining the effective features such as the signal segment, TFR, intrinsic mode function, or differential entropy. This data generation method is known as a generative adversarial network (GAN), and it can approximate the feature distribution of raw EEG signals during process training^27)^. While GAN- based methods have been applied in computer vision for various purposes, such as generating images from text^28)^, generating videos with scene dynamics^29)^, and translating from image to image^30)^, this novel method has been implemented in the field of BCI to improve the BCI system performance.

**Figure 1:**
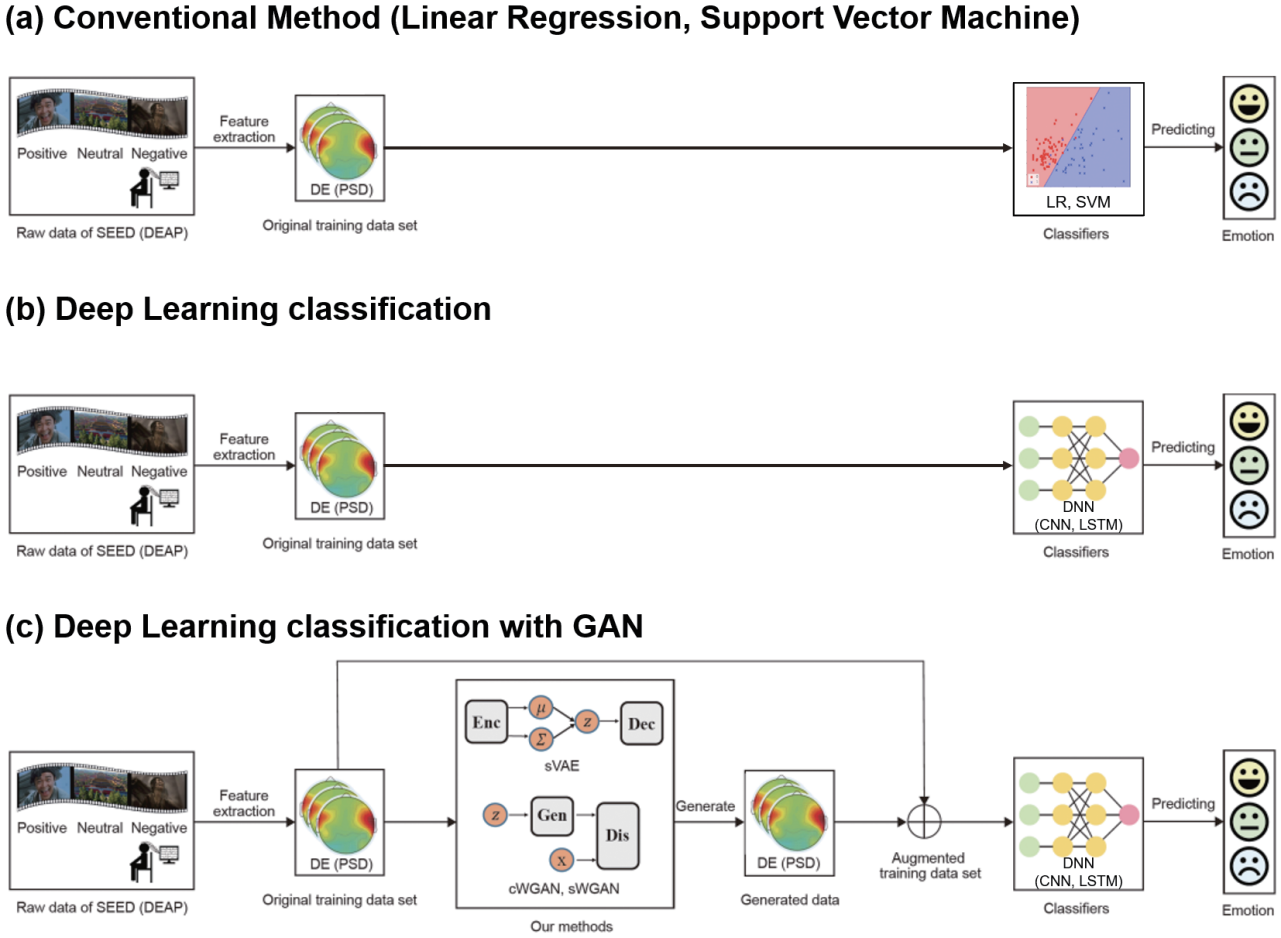
Concepts of classification methods in BCI. For emotion classification based on BCI signal, first, power spectral density (PSD), and differential entropy (DE) features are extracted from the EEG-based emotion recognition dataset. (a) Conventional method classifies emotions using a conventional classifier linear regression (LR) and support vector machine (SVM). (b) Deep learning methods include classifiers such as the convolutional neural network (CNN) and long short-term memory (LSTM). (c) Deep learning method is combined with a generative adversarial network (GAN). GAN generates realistic data and augments the original training dataset. Finally, the performance of BCI systems using SVM and DNN with shortcut layers is evaluated.

For example, Roy et al.^31)^ leveraged the original version of GAN for BCI to classify trials based on the left- and right-hand motor imagery. The time- frequency characteristics of real and artificial EEG signals were compared using the short-term Fourier transform and Welch's power spectral density for evaluation. The results showed that GANs can capture important features of motor imagery EEG data, such as power variations, and that the power variation between the raw and artificially generated EEG signals was in the same frequency bin of Welch's power spectral density. Pascual et al.^32)^ used conditional Least Squares GAN (LSGAN) to alert caregivers and reduce the impact of seizures on patients' quality of life for epilepsy manifested by recurrent unprovoked seizures. LCGAN generated synthetic seizure-like EEG signals to train seizure detection and subsequently improved the detection performance by 1.2% overall relative to training only with real samples. Furthermore, Zhang et al. proposed a conditional deep convolutional GAN (cDCGAN) method for generating a large number of artificial EEG signals for data augmentation to improve CNN performance and overcome the problems associated with small training datasets. For the CNN model, the raw EEG signal was transformed into TRF to learn the time-frequency features from the TFR of the raw EEG signal using a two-dimensional kernel. Thus, cDCGAN is used to generate artificial TFR from the EEG signal and subsequently inverse the wavelet transform to generate waveform EEG signal. Therefore, data augmentation based on cDCGAN improved the classification accuracy of motor imagery tasks of the left- or right-hand movements from 82.8% to 85.8%^33)^. Furthermore, the proposed methods were^34)^ based on two deep generative models (variational autoencoder (VAE) and GAN) and two augmentation strategies (Figure 1). The full usage strategy appended all generated data to the training dataset without judging the quality of the generated data, whereas partial usage selected only high-quality data and appended the data to the training dataset. These three methods are known as conditional Wasserstein GAN (cWGAN), selective VAE (sVAE), and selective WGAN (sWGAN). The effectiveness of these models was evaluated through a systematic experimental study on two public EEG datasets for emotion recognition, such as SEED and DEAP. First, realistic EEG training data were generated in two forms, such as power spectral density and differential entropy. Subsequently, the original training datasets were augmented to generate a different number of realistic-like EEG data. Finally, SVM and deep neural networks (DNN) with short cut layers were trained to develop an effective model using the original and augmented training datasets. Therefore, augmented training datasets by sWGAN enhance the performance of EEG-based emotion recognition models from 83.3% to 92.2% (i.e., 10.2% improvement) and outperform existing data augmentation methods such as cWGAN (from 83.3% to 90.7%), sVAE (from 83.3% to 80.6%), Gaussian noise (from 83.3% to 85.8%), and rotational data augmentation (from 83.3% to 75.7%). Thus, augmentation based on GAN can improve BCI system performance while reducing the cost of acquiring EEG signal data and the effort of medical experts and patients.

## Conclusion and Future Directions

This paper summarizes the developments made in brain-computer interfaces in the last decade to investigate the current trends in BCI research in the fields of medicine, communication, and control for clinical applications. We have summarized the challenges and limitations of the current BCI systems and proposed potential solutions. Numerous BCI systems have been developed, gradually progressing from a simple statistical model to a deep learning model, and consequently, BCI performance has improved over time. However, achieving a classification accuracy of > 90% via an online approach is still difficult. Thus, further development is required for implementing medical BCI systems on medicine settings. Furthermore, individual BCI systems require training and parameter tuning for each task to be completed and they lack versatility. Considering the numerous clinical tasks to be achieved for clinical applications, developing a BCI system for each task is not feasible. To overcome this versatile problem, the Global Workspace Theory^35)^, which refers to a large-scale system integrating and distributing information among networks of specialized modules to create higher-level forms of cognition and awareness, has recently attracted considerable research attention, further advancing deep learning. Unsupervised neural translation between multiple latent spaces (neural networks trained for distinct tasks on distinct sensory inputs and/or modalities) to create a unique, amodal Global Latent Workspace can lead to improvement in the versatility and performance of BCI systems; this may be a promising theory in the medical BCI field^36)^.

## Funding

This research was supported by Brain/MINDS Beyond program (grant no. JP21dm0307101) of the Japan Agency for Medical Research and Development (AMED), AMED under grant number JP22wm0425006, JSPS KAKENHI under grant number JP21K07690 and 21K15851, a Grant-in-Aid for Special Research in Subsidies for ordinary expenses of private schools from The Promotion and Mutual Aid Corporation for Private Schools of Japan, and the Juntendo Research Branding Project. This work was also supported by JST, Moonshot R&D Grant Number JPMJMS2012.

## Author contributions

YS, KK, and RK conceived the presented idea. YS developed the theory and investigated the report on BCI system. KK and RK encouraged YS to investigate this work. All authors discussed the results and contributed to the final manuscript. Finally, all authors read and approved the final manuscript.

## Conflicts of interest statement

RK is an employed at Araya Inc. (Tokyo, Japan). The remaining authors declare that the research was conducted in the absence of any commercial or financial relationships that could be construed as a potential conflict of interest.
